# Case report: Refractory intestinal Behçet’s syndrome successfully treated with tofacitinib: A report of four cases

**DOI:** 10.3389/fimmu.2022.981502

**Published:** 2022-09-15

**Authors:** Na Zhao, Yanchun Tang, Shaokun Wang, Liping Cui, Xuehui Sun, Zhihua Wang, Ying Liu

**Affiliations:** ^1^ Department of Rheumatology, The Affiliated Yantai Yuhuangding Hospital of Qingdao University, Yantai, China; ^2^ Department of Gastroenterology, The Affiliated Yantai Yuhuangding Hospital of Qingdao University, Yantai, China

**Keywords:** Behçet’s syndrome, intestinal diseases, tofacitinib, treatment, case report

## Abstract

Behçet’s syndrome (BS) is a chronic form of relapsing multisystem vasculitis, characterized by recurrent oral and genital ulcers. Intestinal BS is a special type of BS. Volcano-shaped ulcers in the ileocecum are a typical finding of intestinal BS, and punched-out ulcers can be observed in the intestine or esophagus. At present, there is no recognized radical treatment for intestinal BS. Glucocorticoids and immunosuppressants are currently the main drugs used to improve the condition. Although it has been reported that monoclonal anti-TNF antibodies may be effective for some refractory intestinal BS, further randomized, prospective trials are necessary to confirm these findings. Some patients are restricted from using biological agents because of serious allergic reactions of drugs, inconvenient drug injections or the impact of the novel coronavirus epidemic. If endoscopic remission (endoscopic healing) is not achieved for a prolonged period of time, serious complications, such as perforation, fistula formation, and gastrointestinal bleeding can be induced. Therefore, it is necessary to develop new treatment methods for controlling disease progression. We reviewed the relevant literature, combined with the analysis of the correlation between the pathogenesis of BS and the mechanism of Janus kinase (JAK) inhibition, and considered that tofacitinib (TOF) may be effective for managing refractory intestinal BS. We report for the first time that four patients with severe refractory intestinal BS were successfully treated with TOF. We hope to provide valuable information on JAK inhibitors as potential therapeutic targets for the treatment of severe refractory intestinal BS.

## Introduction

Gastrointestinal (GI) involvement is a serious complication of Behçet’s syndrome (BS). Early diagnosis of intestinal BS is difficult because of the lack of specificity in the clinical manifestations of the digestive system and the lack of specific autoantibodies for diagnosis. Therefore, when patients with BS have digestive system symptoms such as abdominal pain, diarrhea, bloody stool, and constipation, endoscopy should be performed as soon as possible to confirm the diagnosis of intestinal BS (1). Simultaneously, NSAID ulcers, inflammatory bowel disease, tuberculosis and other infectious diseases should be excluded as potential causes of these symptoms (2). For patients with intestinal BS who have achieved the disappearance of clinical symptoms and normalization of C-reactive protein (CRP) levels, endoscopic remission (endoscopic healing) is the ultimate treatment goal (3). The response of some patients to traditional treatments is not ideal. Failure to achieve endoscopic remission (endoscopic healing) may lead to serious complications that can seriously affect patients’ quality of life.

At present, the exact pathogenesis of intestinal BS remains unknown, but it has been found that T cell immune dysfunction, particularly the activation of helper (Th)1/Th17 cells, the weakening of regulatory T cells (Treg), and the overexpression of proinflammatory cytokines, is considered to be the cornerstone of BS (4, 5). Tofacitinib (TOF), a JAK1/3 inhibitor targeting T-cell signal transduction, inhibits the signal transducer and activator of transcription 1 (STAT1), T-bet phosphorylation, and differentiation of Th1 and Th17 cells (6). TOF has been successfully used in some cases of BS with refractory uveitis and vessel/cardiac involvement (7–9).

Here we report, for the first time, four patients with severe refractory intestinal BS who did not respond well to traditional treatment and could not achieve endoscopic remission (endoscopic healing). However, after TOF treatment, the treatment goal was achieved.

## Reports

**Figure 1:**
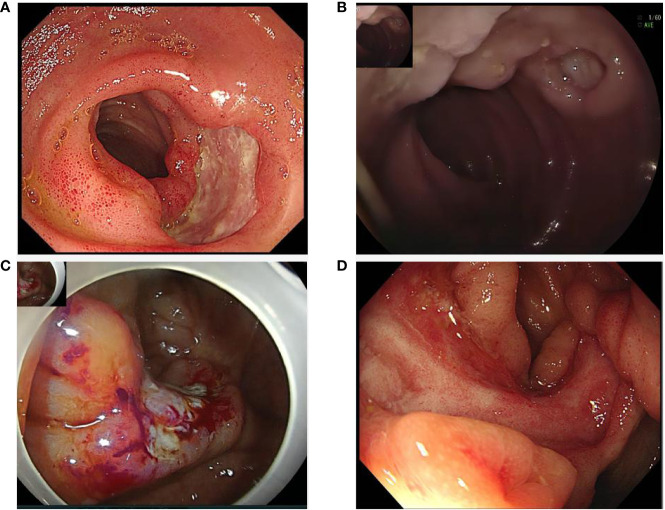
Intestinal ulcers in four patients with intestinal Behçet’s Syndrome before tofacitinib administration: **(A)** Ileocecal ulcer in case 1; **(B)** Ileum ulcer in case 2; **(C)** Ileocecal ulcer in case 3; **(D)** Ileocecal ulcer in case 4.

**Figure 2:**
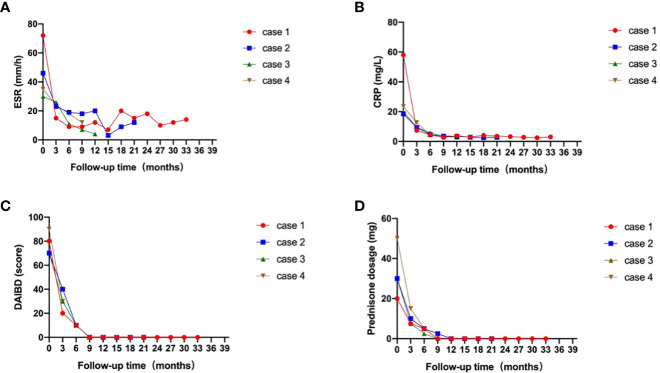
Efficacy of tofacitinib for patients with intestinal Behçet’s syndrome (red: case 1; blue: case 2; green: case 3; brown: case 4). **(A)** Changes in erythrocyte sedimentation rate (ESR); **(B)** Changes in C-reactive protein (CRP); **(C)** Changes in disease activity index of intestinal Behçet’s disease (DAIBD); **(D)** Changes in prednisone dosage, during tofacitinib treatment.

Clinical characteristics and therapeutic interventions for refractory intestinal BS are shown in **Table 1**. The intestinal ulcers in four patients with intestinal BS before TOF administration are shown in **Figure 1**. The efficacy of TOF in the treatment of intestinal BS is shown in **Figure 2**, including changes in the erythrocyte sedimentation rate (ESR), CRP, the disease activity index of intestinal Behçet’s disease (DAIBD), and prednisone dosage during TOF treatment.

**Table 1:** Clinical characteristics and therapeutic interventions of refractory intestinal Behçet’s Syndrome.

Case	Sex	Age	Intestinal BS course	Clinical features	Previous treatment	TOF combined therapies	Present therapies	Follow-up	Clinical response
		(yrs)	(mths)					(mths)	
1	M	67	8	O,G,GI ulcers (terminal ileum,ilececal valve,colon)	CS,SASP,MTX,THD,IFX	CS,SASP,THD	TOF 5mg daily,SASP	33	success
2	F	20	7	O,G,S,GI ulcers (ileum,terminal ileum)	CS,AZA,SASP,CTX,THD	CS,SASP,THD	TOF 5mg daily,SASP	20	success
3	F	49	8	O,G,arthralgia,GI ulcers (ileocecum)	CS,THD,AZA,SASP,CTX	CS,SASP	TOF 5mg two times a day,SASP	12	success
4	F	31	18	O,G,GI ulcers (ileocecum)	CS,THD,CTX,SASP	CS,SASP,THD	TOF 5mg two times a day, Pred 2.5mg daily,SASP	10	success

AZA, azathioprine; BS, Behçet’ Syndrome; CS, corticosteroids; CTX, cyclophosphamide; EN, erythema nodosum; G, genital ulcer; GI, gastrointestinal; IFX, infliximab; MTX, meth-otrexate; O, oral ulcer; SASP, salazosulfapyridine; THD, thalidomide; TOF, tofacitinib.

The procedure followed in this study met ethical standards and was approved by the ethics committee of Yantai Yuhuangding Hospital (2019372). Informed consent was obtained from the patients for all clinical data, information collection, and drug application, as well as for publication of this case report.

### Case 1

A 67-year-old man developed recurrent aphthous ulcers in his mouth starting at the age of 30 years and recurrent penile ulcers starting at the age of 59 years. The patient did not undergo any systematic treatment. On December 8, 2018, he was admitted to a local hospital because of repeated right lower abdominal pain for 8 months and aggravation after eating. Laboratory test results were as follows: fecal occult blood test, positive; hemoglobin (HGB), 96 g/L; ESR, 65 mm/h; CRP, 113.3 mg/L; no abnormality was found in infection, tumor, or immunological indexes. Paired test results were positive. Enteroscopy revealed ulcers in the terminal ileum, ileocecal valve, and colon, which were characterized by large, deep, oval ulcer surfaces, and clear boundaries (**Figure 1A**). The pathological results of the ulcers showed that the mucosa had severe acute and chronic inflammation accompanied by erosion, inflammatory exudation, granulation tissue formation, the infiltration of inflammatory cells in the blood vessel wall and the occlusion of blood vessels. No abnormalities were found on abdominal imaging. The DAIBD score was 90. The patient was treated with prednisone (50 mg PO daily), salazosulfapyridine (SASP, 1.0 g po two times a day), and thalidomide (THD, 50 mg PO per night). His abdominal pain was relieved.

After 4 months, the prednisone dose was gradually decreased to 15 mg daily. However, on April 25, 2019, enteroscopy showed that the large ileocecal ulcer had not healed. The treatment regimen was adjusted to prednisone (60 mg po daily) combined with methotrexate (MTX, 12.5 mg po once a week), SASP, and THD. After 4 months of treatment, the abdominal pain recurred when prednisone was reduced to 15 mg daily. On August 10, 2019, enteroscopy revealed that the ulcer had not healed. The patient was hospitalized in our department on August 20, 2019, for further treatment. Other causes of intestinal ulcers were also excluded from this study. ESR was 72mm/h. CRP was 58mg/L. The DAIBD score was 80. The diagnosis of refractory intestinal BS was clear. The patient developed a generalized itchy rash during infliximab (IFX) treatment. The patient then received TOF (5 mg PO twice a day) and prednisone (20 mg PO daily) combined with SASP and THD. After 4 months of treatment, enteroscopy revealed that the ileocecal ulcer had completely healed. In June 2020, prednisone was gradually reduced to zero. The TOF was reduced to 5 mg/day. To date, the patient’s DAIBD score, ESR, and CRP level have been normal (**Figure 2**).

### Case 2

A 20-year-old woman was hospitalized in the digestive department on February 24, 2020, because of abdominal pain and diarrhea for 3 days and bloody stool for 11 hours. The history of the present illness was further investigated. The patient experienced intermittent pain and discomfort in the right lower abdomen without obvious induction 7 months prior and recurrent aphthous ulcer in the mouth and vulva 6 months prior. More than two months ago, nodular erythema recurred on both sides of the shank without induction. In the past two months, the weight dropped by 2.5 kg. Laboratory test results were as follows: fecal occult blood test, positive; HGB, 78 g/L; ESR, 46 mm/h; CRP, 38.5 mg/L. No abnormalities were found in the stool bacterial culture, infection, tumor, or other immunological indices. Paired test results were positive. Abdominal CT revealed a suspected incomplete intestinal obstruction. On February 25, 2020, enteroscopy showed scattered multiple round ulcers of approximately 0.6-2.0 cm that could be seen in the ileum, which were characterized by white moss and obvious edema of the surrounding mucosa (**Figure 1B**). The biopsy results of the large ulcers showed that the acid-fast bacilli microscopic examination and Mycobacterium tuberculosis rpoB gene examination were negative, and the pathology showed acute and chronic mucosal inflammation.

On March 4, 2020, the patient was transferred to the rheumatology department for further treatment. She was diagnosed with severe intestinal BS, with a DAIBD score of 105. The patient was treated with prednisone (30 mg PO daily), azathioprine (ASA, 100 mg PO daily), and SASP. Two weeks later, AZA was stopped due to a decrease in leukocyte levels, which was switched to cyclophosphamide (CTX, 100 mg PO once every other day) after the recovery of leukocyte levels. After 3 months, prednisone was gradually decreased to 10 mg daily. Although the oral ulcer, vulvar ulcer, and nodular erythema significantly improved, the pain persisted in the right lower abdomen. Therefore, the treatment combined with thalidomide was based on the original treatment.

In September 2020, enteroscopy revealed that the ileal ulcer had not healed. Therefore, CTX was discontinued and the patient was switched to TOF (5 mg PO twice a day). Three months later, enteroscopy revealed that the ileal ulcer had healed. In March 2021, the prednisone dose was gradually reduced to zero. In July 2021, TOF was adjusted to 5 mg daily. THD was discontinued in October 2021. To date, the patient’s DAIBD score, ESR, and CRP level have been in remission (**Figure 2**).

### Case 3

A 49-year-old woman presented with recurrent aphthous ulcers in the oral cavity and vulva 7 years ago and repeated right lower abdominal pain 6 years ago, accompanied by multiple joint pain but no swelling. After administering THD alone, the frequency of oral and vulvar ulcers decreased significantly, but intermittent abdominal pain persisted. In June 2019, the patient’s abdominal pain suddenly worsened, accompanied by diarrhea, with watery stools ranging from 3 to 5 times a day without mucus and pus. The patient was hospitalized on June 26, 2019. In the past year, the patient’s weight had dropped by 4 kg. Laboratory test results were as follows: fecal occult blood test, positive; HGB, 114 g/L; ESR, 50 mm/h; CRP, 35.86 mg/L. Examinations were performed to exclude infections, tumors, and other rheumatic diseases. Paired test results were positive. PPD test was negative. Abdominal enhanced CT showed no obvious abnormalities. Enteroscopy revealed an irregular ulcer in 1/2 of the ileocecal cavity, which was characterized by white scar-like changes on the surface, erosion and necrosis, and white moss at the bottom (**Figure 1C**). Acute and chronic inflammation of the ileocecal region with erosion was observed under pathological examination. The patient’s DAIBD score was 90.

The patient was treated with prednisone (40 mg PO daily), AZA (100 mg PO daily), and SASP. After 4 months, prednisone was reduced to 10 mg daily, and the patient still experienced mild discomfort in the right lower abdomen. In October 2019, enteroscopy revealed that the ileocecal ulcer had not healed. The prednisone dosage was adjusted to 30 mg daily. ASA was stopped and switched to CTX (100 mg PO once every other day).

In February 2021, the patient was admitted to the emergency department because of aggravation of abdominal pain. The ESR was 30 mm/h, and the CRP level was 20 mg/L. Further examination was performed to rule out other causes of the ulceration. Enteroscopy revealed the continued presence of the previously noted ileocecal ulcer. The patient’s DAIBD score was 80. On February 24, 2021, the patient was treated with prednisone (30 mg PO daily), TOF (5 mg PO twice daily), and SASP. After two weeks, the patient’s abdominal pain gradually subsided, and inflammatory indices gradually decreased. After 5 months, enteroscopy revealed that the ileocecal ulcer had healed. Nine months later, the prednisone dose was gradually reduced to zero. To date, the patient continues to be in remission (**Figure 2**).

### Case 4

A 31-year-old woman developed recurrent aphthous ulcers in her mouth starting at the age of 16 years and recurrent vulvar ulcers starting at the age of 21 years. She had been treated with small doses of prednisone and THD for a short period, but the ulcers recurred after withdrawal. In December 2020, she was admitted to the digestive department of a local hospital because of abdominal pain. Laboratory test results were as follows: ESR, 32 mm/h; CRP, 15 mg/L. Endoscopy revealed an ulcer in the ileocecal region. Pathology showed acute and chronic inflammation accompanied by tissue necrosis, inflammatory cell infiltration, small vessel proliferation, no acid-fast bacilli, and no tumor cells. The patient was diagnosed with intestinal BS.

The patient was treated with prednisone (50 mg/day) and SASP. After 3 months, the prednisone dose was reduced to 20 mg daily. Owing to persistent abdominal pain, she was treated with CTX (100 mg PO once every other day). The patient’s abdominal symptoms were not significantly relieved. The patient was admitted to our hospital in August 2021 owing to aggravation of abdominal pain. She had lost 4 kg in the past two months. Laboratory test results were as follows: fecal occult blood test, positive; HGB, 86 g/L; ESR, 35 mm/h; CRP, 23.4 mg/L. Tuberculosis and the tumor were ruled out. Abdominal CT showed thickening and roughness of the ileocecal wall, which was considered an inflammatory change. On August 17, 2021, enteroscopy showed that an ulcer with a size of about 4.0 cm x 1.2 cm in the ring 2/3 cavity was found near the ileocecal valve, which was characterized by white scar in the local mucosa, congestion and edema in the surrounding mucosa (**Figure 1D**). Pathological examination revealed acute and chronic inflammation of the mucosal tissue. The patient’s DAIBD score was 80.

The patient was treated with prednisone (50 mg PO daily), TOF (5 mg PO twice a day), SASP, and THD (discontinuation due to obvious reduction in menstrual volume). After two weeks, the abdominal pain gradually subsided. Subsequently, the prednisone dose was gradually reduced to 2.5 mg daily. In December 2021, enteroscopy revealed that the ileocecal ulcer had healed, and the patient’s DAIBD score, ESR, and CRP levels were normal (**Figure 2**).

## Discussion

This paper reports four cases of patients with refractrory intestinal Behçet’s syndrome characterised by obstinate peptic ulcers and sustained gastrointestinal symptoms, successfully treated with Tofacitinib. It has been reported that some BS patients without gastrointestinal symptoms can have peptic ulcers on endoscopy (10). Therefore, once patients with BS have gastrointestinal symptoms, gastrointestinal endoscopy should be performed as soon as possible to determine the diagnosis of intestinal BS, which can help identify peptic ulcer early and permit clinicians to intervene in time to reduce the occurrence of complications.

Behçet’s syndrome (BS), also known as Behçet’s disease (BD), is characterized by a concurrence of innate and adaptive immune disorders and is considered to be an intermediate between the innate (autoinflammation) and adaptive (autoimmunity) immune disease (11, 12). The main manifestations are recurrent oral ulcers, genital ulcers, uveitis, and skin damage, as well as damage to peripheral blood vessels, heart, nervous system, gastrointestinal tract, joints, lungs, and kidney (13, 14). BS is classified as variant vasculitis in the nomenclature of the Chapel Hill Consensus Conference(CHCC) vasculitis revised in 2012 (15).

Intestinal BS is a special form of BS. The incidence of intestinal BS varies from 4% to 38% (16). The age of onset is usually in the range of 15–50 years of age, and the incidence rate is similar between men and women. Among the four patients described in this report, three were young and middle-aged women and one older male. The entire digestive tract can be affected, from the esophagus to the anus, particularly the terminal ileum, ileocecum, and ascending colon (17). The clinical manifestations of gastrointestinal tract involvement also vary, including abdominal pain, abdominal mass, diarrhea, abdominal distension, dysphagia, vomiting, bloody stools, and constipation (18). Severe cases may be complicated by peptic ulcers, bleeding, intestinal perforation, intestinal obstruction, and fistula formation. Due to the lack of specific clinical manifestations and autoantibodies in patients with intestinal BS, the diagnosis is often delayed.

Typically, volcano-shaped ulcers around the ileocecal region are observed in intestinal BS; these are characterized by round or oval shape (number ≤ 5), clear boundaries, diameter greater than 1 cm, and deep wounds. CT findings of the intestinal tract may show thickening of the intestinal wall and peri-intestinal infiltration shadow, and some may show mesenteric vascular congestion, fistula formation, and surrounding adipose tissue turbidity (3), such as in cases 2 and 4.

Pathological manifestations are nonspecific manifestations of acute and chronic inflammation of the intestinal mucosa. Gastrointestinal manifestations usually occur 4.5–6 years after the onset of oral ulcerations. However, intestinal lesions can sometimes precede extra-intestinal manifestations (19). The four cases described in this report all started with oral and/or genital ulcers, and intestinal involvement occurred months or years later. Many BS patients do not pay attention to oral ulcers at the early stage of onset, and then later visited a doctor because of severe genital ulcers or severe abdominal pain. Therefore, some patients with intestinal BS first visit the digestive department. When a patient has typical volcano-shaped ulcers in the ileocecal region, the doctor needs to carefully ask for the patient’s past medical history to ensure accurate diagnosis and treatment.

According to the 2014 international diagnostic criteria for BS (1), all four patients described in this report were diagnosed with BS. The diagnosis of intestinal BS was confirmed using gastrointestinal symptoms and endoscopy. The evaluation of intestinal BS includes clinical symptoms, HGB, ESR, CRP, endoscopy, and DAIBD score, which have guiding significance for the evaluation of treatment effects (20). Endoscopic remission (endoscopic healing) is the most important aim in patients with intestinal BS. All four patients with intestinal BS in this report had a DAIBD score of more than 75 before initial treatment, which was defined as severe intestinal BS. After they were diagnosed with intestinal BS, they were all treated with medium to large doses of glucocorticoids combined with a variety of immunosuppressants; however, the patient’s abdominal pain recurred repeatedly, and the intestinal ulcer did not heal for a long time. The patients were thus diagnosed with refractory intestinal BS.

At present, there is no radical cure for intestinal BS. The evidence for effective treatment of intestinal BS mainly depends on retrospective observational data, and there are few controlled clinical studies. The purpose of treatment is to induce and maintain relief of gastrointestinal symptoms, promote mucosal healing, reduce recurrence, and avoid surgical treatment and irreversible intestinal injury. The EULAR guidelines suggest that patients with moderate to severe intestinal BS require glucocorticoids combined with immunosuppressants (2). Patients with refractory intestinal BS generally respond poorly to traditional treatment, and TNF inhibitors (infliximab or adalimumab) can be considered (2, 15, 21, 22). However, owing to serious adverse reactions and inconvenient drug injection or the influence of novel coronavirus, some patients are restricted from using biological agents. Moreover, the persistent failure to achieve endoscopic remission (endoscopic healing) can lead to serious complications. Therefore, it is necessary to develop new treatment methods for controlling disease progression.

At present, there have been some cases of successful treatment of refractory BS eye disease and vessel/cardiac involvement with TOF. Liu et al. reported that six patients with refractory intestinal BS received TOF treatment; the intestinal ulceration healed in one patient and persisted in the other five patients (7). However, three of those patients had very serious complications, such as fistula formation or perforation. The four patients described in this report with refractory intestinal BS (without perforation and fistula formation) achieved good results after TOF treatment, including relief of clinical symptoms, reduction of inflammatory indicators, gradual healing of intestinal ulcers, and gradual withdrawal of glucocorticoids in some patients. Therefore, we suggest that the early addition of TOF to patients with refractory intestinal BS with persistent nonhealing of intestinal ulcers after active traditional drug treatment may be a better option for those patients who do not have severe complications such as perforation or fistula.

Thus, tofatinib may be a potential treatment for refractory intestinal BS. The Janus kinase/signal translator and activator of transcription (JAK-STAT) signaling pathway and its corresponding cytokines have been implicated in the pathogenesis of BS (5). TOF can inhibit JAK, STAT1, and T-bet phosphorylation, which block the signaling of interleukin (IL)-2, IL-4, IL-6, IL-23, interferon (IFN -γ), and IFN -α, and suppress the differentiation of Th1 and Th17 cells (6). Transcriptome analysis of patients with BS demonstrates that Th17 related genes and type I IFN-inducible genes are upregulated, and that JAK/STAT signaling promotes the activation of Th1/Th17 cytokines (23).

TOF has been approved for the treatment of rheumatoid arthritis (RA), ankylosing spondylitis (AS), psoriatic arthritis (PsA), ulcerative colitis (UC) and other inflammatory diseases (24). These diseases and BS share some common clinical features and genetic variants (24). For example, they share the same genetic background as the JAK/STAT-activated cytokines IL-23R and IL-21R (25). The four patients with refractory intestinal BS described in this report did not respond well to traditional treatment, and one of the patients had adverse reactions after receiving IFX. After receiving TOF treatment, all patients achieved disappearance of clinical symptoms, normalization of CRP levels, and endoscopic remission. Among them, glucocorticoids were gradually discontinued in three patients. The TOF dose was halved in two patients. We suggest that TOF had a definite effect on refractory intestinal BS in these four patients.

In conclusion, considering the key role of abnormal activation of T cells in the pathogenesis of BS, the JAK/STAT signaling pathway may be a potential target for the treatment of patients with refractory intestinal BS. JAK inhibitors are potential treatments for refractory intestinal BS.

This case report has limitation. The number of cases reported in this case report is relatively small, including only 4 patients with refractory intestinal BS. Although TOF is effective in these four patients with refractory intestinal BS, however, we will increase the number of clinical applications in future work, further evaluate the clinical efficacy of TOF in the treatment of refractory intestinal BS, and conduct multi-center research to confirm the efficacy and safety of JAK inhibitors.

## Data availability statement

The original contributions presented in the study are included in the article/supplementary material. Further inquiries can be directed to the corresponding author.

## Ethics statement

The studies involving human participants were reviewed and approved by the ethics committee of Yantai Yuhuangding Hospital (2019372). The patients/participants provided their written informed consent for the publication of any potentially identifiable images or data presented in the article.

## Author contributions

NZ performed data analysis and wrote the manuscript. YT, SW, LC, and XS provided clinical information. ZW provided endoscopic images. YL supervised the study. All authors have contributed to the manuscript and approved the submitted version.

## Funding

This work was supported by Yantai Yuhuangding Hospital’s Youth Scientific Research Startup Fund Project (202017).

## Conflict of interest

The authors declare that the research was conducted in the absence of any commercial or financial relationships that could be construed as a potential conflict of interest.

## Publisher’s note

All claims expressed in this article are solely those of the authors and do not necessarily represent those of their affiliated organizations, or those of the publisher, the editors and the reviewers. Any product that may be evaluated in this article, or claim that may be made by its manufacturer, is not guaranteed or endorsed by the publisher.
